# The Application of DVDMS as a Sensitizing Agent for Sono-/Photo-Therapy

**DOI:** 10.3389/fphar.2020.00019

**Published:** 2020-02-07

**Authors:** Bingjie Mai, Xiaobing Wang, Quanhong Liu, Kun Zhang, Pan Wang

**Affiliations:** National Engineering Laboratory for Resource Development of Endangered Crude Drugs in Northwest China, The Key Laboratory of Medicinal Resources and Natural Pharmaceutical Chemistry, The Ministry of Education, College of Life Sciences, Shaanxi Normal University, Xi'an, China

**Keywords:** sinoporphyrin sodium, sonodynamic therapy, photodynamic therapy, photodynamic antimicrobial therapy, nanomaterials

## Abstract

Both photodynamic therapy (PDT) and sonodynamic therapy (SDT) are fast growing activated therapies by using light or ultrasound to initiate catalytic reaction of sensitizing agents, showing great potentials in clinics because of high safety and noninvasiveness. Sensitizers are critical components in PDT and SDT. Sinoporphyrin sodium (DVDMS) is an effective constituent derived from Photofrin that has been approved by FDA. This review is based on previous articles that explore the applications of DVDMS mediated photodynamic/sonodynamic cancer therapy and antimicrobial chemotherapy. Researchers utilize different cell lines, distinct treatment protocols to explore the enhanced therapeutic response of neoplastic lesion. Moreover, by designing a series of nanoparticles for loading DVDMS to improve the cellular uptake and antitumor efficacy of PDT/SDT, which integrates diagnostics into therapeutics for precision medical applications. During the sono-/photo-activated process, the balance between oxidation and antioxidation, numerous signal transduction and cell death pathways are also involved. In addition, DVDMS mediated photodynamic antimicrobial chemotherapy (PACT) can effectively suppress bacteria and multidrug resistant bacteria proliferation, promote the healing of wounds in burn infection. In brief, these efficient preclinical studies indicate a good promise for DVDMS application in the activated sono-/photo-therapy.

## Introduction

According to the World Health Organization's latest global cancer data, the cancer burden has risen to 18.1 million new cases and 9.6 million deaths by 2018. Global patterns show that nearly half of the new cases and more than half of the cancer deaths occur in Asia (Data, 2018). From the overall trend, the number of cancer patients and the number of deaths in the world will continue to grow, and the situation in China is becoming more and more serious.

Traditional cancer treatments, i.e., surgical resection, radiation therapy, and chemotherapy have distinct limitations. Due to the expansion of tumor tissue and the root-like infiltration, the lymphatic and blood distant organ metastasis has already occurred when the tumor is diagnosed, so the complete surgical removal would be impossible. Meanwhile, radiotherapy and traditional chemotherapy produce severe side-effects, they cannot selectively kill tumor cells and result in serious damage to the immune system (Miller et al., 2016). Moreover, the current therapies are very expensive, which could be a fatal blow to the whole family and cause many patients give up treatment because they cannot afford the high treatment price. Many researchers are trying to develop new strategies to combat cancer. Among the newly emerged cancer therapies, the activated therapy using light or ultrasound as stimulus for stimulating sensitizers, and the derived photodynamic therapy (PDT) and sonodynamic therapy (SDT) will bring new hope to improve the life time and quality of cancer patients (Kenyon et al., 2009).

The first generation sensitizer Photofrin^®^ belongs to one kind of porphyrin derivatives, it has efficient singlet oxygen production and been approved by FDA for treating cancers *via* PDT (AB and HS, 2013). Although Photofrin^®^ has achieved positive therapeutic effects in clinic, there are still many shortcomings, such as complex components, unsatisfactory spectrums, and systemic dark toxicities (O'Connor et al., 2010). One of the important reasons is that Photofrin^®^ is a mixture of unclear porphyrin components. Sinoporphyrin sodium (DVDMS) is an effective constituent based on Photofrin^®^ (Hu et al., 2015). DVDMS has 98.7% chemical purity and is highly soluble in water, resulting in relatively short-term skin sensitivity and high potential of singlet oxygen yield. Studies indicate the photosensitivity of DVDMS is 10 times higher than that of Photofrin^®^ (Wang et al., 2015). Besides, the sonoactivity of DVDMS is also much higher than that of Photofrin^®^ and several other porphyrins (Xiong et al., 2015). SDT uses ultrasound to stimulate sonosensitizer that mostly derived from photosensitizers in PDT (Trendowski, 2014). Ultrasound has good biological tissue penetration, and can focus its energy into the specific depth to produce bioeffects in the targeting site (Rosenthal et al., 2004). To some extent, SDT overcomes the limitation of PDT superficial diseases treatment because of the short penetration of light. In addition to the singlet oxygen mechanism in PDT, more complex explanations referring to mechanical stress, cavitational effects, and multiple reactive oxygen species are involved in SDT (McHale et al., 2016). In addition to cancer disease, the spread of multidrug resistant bacteria are another threat to human health, and the excessive abuse of antibiotics has aroused great concerns in recent years (Roy et al., 2016). Photodynamic antimicrobial therapy (PACT) is a promising alternative for the treatment of drug-resistant infections (Wainwright, 1998). Therefore, in this work, we provide a state-of-the-art overview of the applications of DVDMS for sono-/photo-therapy, including DVDMS in antitumor and antibacteria research. In recent studies, researchers have worked closely with advanced nanobiotechnology to investigate the potential of nanoDVDMS in precison theranostics (**Table 1**).

**Table 1:** The application of DVDMS as a sensitizing agent for activated cancer and bacteria therapy.

Treatment method	Targets	*In vitro/In vivo*	Sensitizer	Reference
SDT	K562, U937, Eca109, NIH3T3, SW620, SPL, PBMC Cells	*In vitro*	DVDMS	Anti-Cancer Drugs 2014, 25, 174–182
PDT	Eca-109 Cells	*In vitro*	DVDMS	Photochemistry and Photobiology, 2014, 90 1404–1412
SDT	S180 Cells	*In vitro/In vivo*	DVDMS	Biopharm. Drug Dispos. 35 50–59 (2014)
SDT	Eca-109 Cells	*In vitro*	DVDMS	International Journal of Nanomedicine. 2014:9 3077–3090
PDT	Beagle dogs	*In vivo*	DVDMS	Photochem. Photobiol. Sci.,2015, 14, 815
PDT	4T1 Cells	*In vitro/In vivo*	DVDMS	Theranostics 2015; 5(7) 772-786
SDT	NIH3T3 cells, S180 cells	*In vitro/In vivo*	DVDMS	Scientic Reports. 2015 Dec 3;517485
SPDT	4T1 Cells	*In vitro/In vivo*	DVDMS	Ultrasonics Sonochemistry 31 (2016) 437–448
PDT	MDA-MB-231 Cells	*In vitro*	DVDMS	Photodiagnosis and Photodynamic Therapy 13 (2016) 58–65
PDT	4T1 Cells	*In vivo*	DVDMS	Journal of Photochemistry & Photobiology, B Biology 160 (2016) 299–305
SDT	U87 MG-Red-FLuc human glioblastoma	*In vitro/In vivo*	DVDMS	Ann Biomed Eng. 2018 Oct 12
PDT	Eca-109 Cells	*In vitro*	DVDMS	Photodiagnosis and Photodynamic Therapy 24 (2018) 198–205
SDT	CT26 Cells	*In vitro/In vivo*	DVDMS+MB	International Journal of Biological Sciences. 2015; 11(12) 1401-1409.
SDT + 2DG	MDA-MB-231, MCF-7, HUVEC, 4T1 Cells	*In vitro/In vivo*	DVDMS	Ultrasound Med Biol. 2018 Jun;44(6)1233-1243.
PDT, Fluorescence imaging	U87MG Cells	*In vitro/In vivo*	GO-PEG-DVDMS	Biomaterials. 2015 February; 42 94–102
PDT/PTT	PC9 Cells	*In vitro/In vivo*	GO-PEG-DVDMS	Nanoscale. 2015 February 14; 7(6): 2520–2526
PDT/PTT	MCF-7 Cells	*In vitro/In vivo*	Mn/DVDMS	Adv Mater. 2017 June; 29(23):
PTT/PDT	4T1 Cells	*In vitro/In vivo*	RGD-ferritins-DVDMS	Biomater. Sci., 2017, 5, 1512
SDT	MDA-MB-231, 4T1 Cells	*In vitro/In vivo*	DVDMS-liposome–microbubble complexes (DLMBs)	Nano Research-s12274-017-1719-8
PDT	MCF-7 Cells	*In vitro/In vivo*	DVDMS-PTX-liposome (PDL)	Biomaterials 141 (2017) 50e62
SDT, MR and fluorescence imaging	U87 human glioma Cells	*In vitro/In vivo*	DVDMS-manganese ions-nanoliposomes (DVDMS-Mn-LPs)	J Cell Mol Med. 2018 Nov;22(11)5394-5405
SDT	U87 MG-Red-FLuc human glioblastoma	*In vitro/In vivo*	DVDMS	Ann Biomed Eng. 2019 Feb; 47(2): 549-562
SDT	H446 Cells	*In vitro/In vivo*	DVDMS	Cell Physiol biochem. 2018; 51(6):2938-2954.
SDT	C6 gliomas Cells	*In vitro/In vivo*	iRGD-Lipo- DVDMS	Biomater Sci. 2019 Jan 2. Doi:10.1039/c8bm01187g.
SDT	Hepatocellular carcinoma (HCC) Cells	*In vitro*	DVDMS	Int J Biochem Cell Biol. 2019 Jan 17.
PDT	Human colorectal cancer (CRC)	*In vitro/In vivo*	DVDMS	Int J Biol Sci. 2019 Jan 1; 15(1):12-23.
PACT	S. aureus	*In vitro*	DVDMS	Lasers in Surgery and Medicine 48400–408 (2016)
PACT	S. aureus, MDR-S. aureus	*In vitro/In vivo*	DVDMS	International Journal of Nanomedicine 201712 5915–5931

## DVDMS Used in Activated Cancer Therapy

### Basic Features of DVDMS

The spectroscopic properties of sensitizers are critical for activated therapies (Dougherty, 1985). Wang et al. measured the characteristic absorption and fluorescence of DVDMS in various pH values and ionic strength (Wang et al., 2015b). The results suggest that DVDMS existed as a monomer in aqueous solution, which was similar to the porphyrin results reported in other literatures. Porphyrin dimers and trimers compounds were unstable as solid power or in solution by Pandey' study (Pandey and Dougherty, 1989). For comparison, Wang and Ni et al. evaluated the safety of DVDMS in the 4T1-bearing mice and beagle dogs, respectively. They did not find any significant signs of toxicity from physical examination, biochemical indicators, or histopathological observations (Ni et al., 2015; Wang et al., 2015b). According to the results, the unobserved negative effect levels is 2 mg/kg in mice and 1 mg/kg in dogs, and DVDMS administration is relatively safe. The photo-/sono-therapy triggered by DVDMS has broad prospects in future clinical transformation.

### DVDMS Accumulation in Tumors

The principle of PDT/SDT is based on the preferential accumulation of sensitizing agent in tumors, and the enhanced cytotoxicity after activation by light or ultrasound (Kolarova et al., 2009; Sadanala et al., 2014). Therefore, PDT/SDT treatment can not only kill tumor cells to the maximum extent, but also minimize adverse effects (Shirasu et al., 2010). *In vitro* studies suggest DVDMS has a preferential uptake in tumor cells compared with normal healthy cell lines (Hu et al., 2014; Xiong et al., 2015). And DVDMS mainly localizes in the mitochondria of tumor cells, which shares the similarity with other porphyrins (Wu et al., 2016), suggesting mitochondria would be a potential target during photo-/sono-therapy. By using the inherent fluorescence of DVDMS, the *in vivo* findings indicate DVDMS distributes high level in tumor as well as in liver and kidney, the retention ratio of tumor to surrounding healthy tissues is above 3 (Wang et al., 2015b). This agrees well with others' investigations, which show that porphyrins may metabolize through liver and kidney and result in high enrichments (Liu et al., 2007; Wang et al., 2007; Li et al., 2014). The possible tumor accumulation could be explained as follows. First, such selective uptake is determined by the microenvironment surrounding the tumor. Many types of tumor cells express a large number of low-density lipoprotein receptors, and sensitizers combined with low-density protein-binding enter tumor cells *via* endocytosis (Jori and Reddi, 1993; Allison et al., 2010). In addition, the pH value in tumors is generally lower than that in most normal tissues, and cell uptake is reported to increase with decreasing pH (Moan et al., 1980). Second, studies have shown that tumor-associated macrophages take up large amounts of porphyrin derivative in tumors (Korbelik et al., 1991; M et al., 1991). Thus, tumor-associated macrophages may be one of the reasons for DVDMS selective absorption. Third, the abnormal structural characteristics of tumor matrix such as leaky vasculature, compromised lymphatic drainage, a high amount of newly generated collagen that bound porphyrins also contribute to the enhanced accumulation of DVDMS in tumors (Musser et al., 1980). Moreover, compared with Hp, PpIX, and photofrin, DVDMS showed much higher intrinsic red fluorescence, indicating that DVDMS has additional advantages in further clinical tumor tracing and imaging (Xiong et al., 2015).

### DVDMS-Based Photo-Therapy

**Figure 1:**
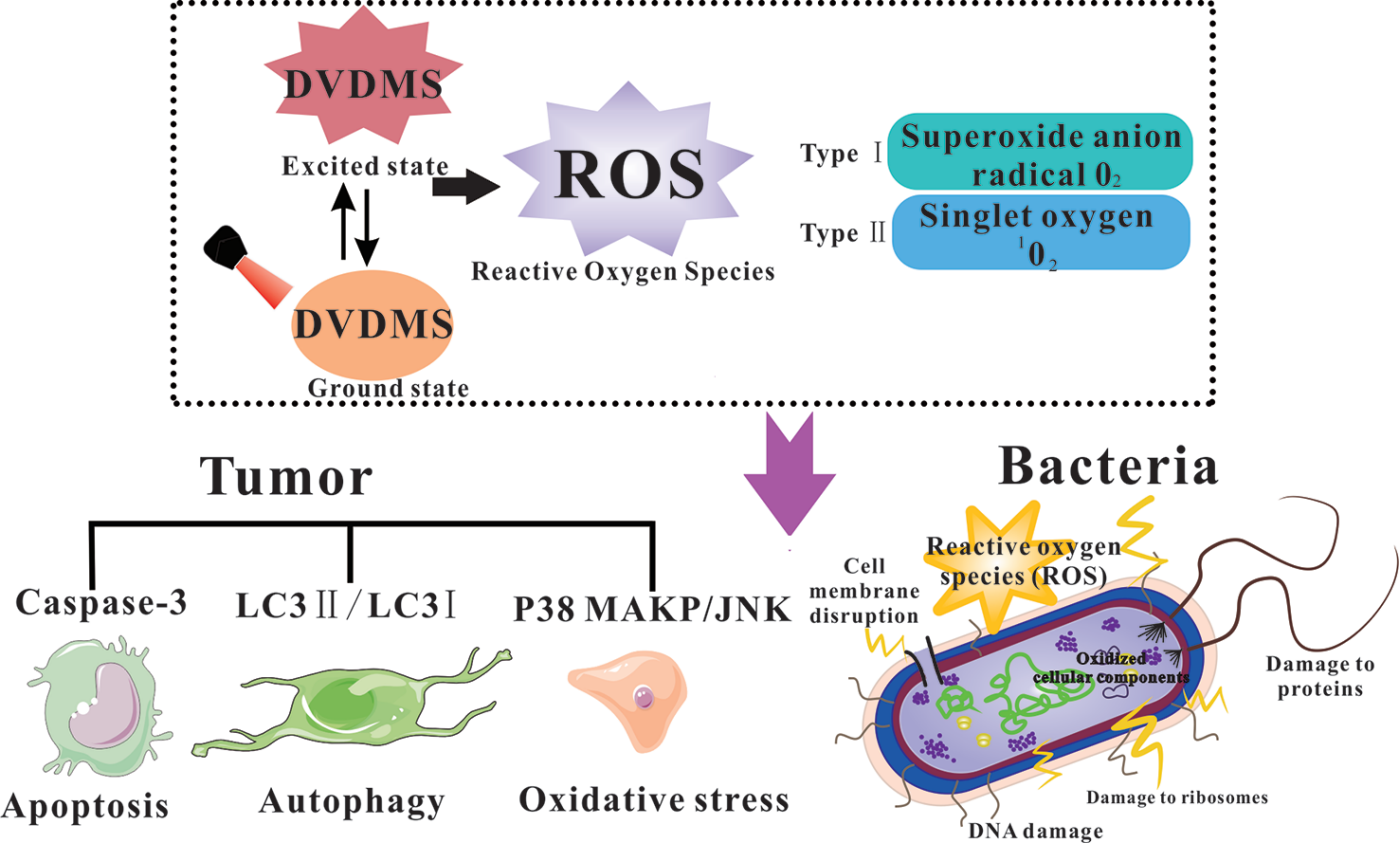
Diagram of ROS generation for photodynamically killing cancer cells and pathogenic microorganisms.

Oxidative damage is the main mechanism of PDT, and the singlet oxygen production is proportional to the effectiveness of PDT (**Figure 1**) (Gorman et al., 2004). Wang et al. utilized the photo-oxidation of DPBF (1, 3-diphenylisobenzofuran) as a quantitative method to evaluate singlet oxygen yield, and the results suggest much higher singlet oxygen production of DVDMS compared with the other porphyrins (Hp, PpIX, and Photofrin) because of its double porphyrin rings (Wang et al., 2015a; Yan et al., 2015).

For direct cell killing, compared to the clinically approved Photofrin^®^, DVDMS at a dose of 1/10 concentration can achieve the same killing effect of Photofrin^®^ (Jiang et al., 2013; Zang et al., 2017a). Intracellular ROS production is considered as the main cause of PDT induced cell death (Allison and Moghissi, 2013). An oxidative stress accompanied with mitochondria dysfunction was involved in DVDMS-PDT (Hu et al., 2015). Apoptosis is a programmed cell death involving protease cascade reactions (Danon et al., 2004). The apoptosis executor Caspase-3 is observed with increasing activity during DVDMS-PDT (Shi et al., 2018). Meanwhile, autophagy plays different roles in different tumor stages and is a vital response to various therapeutic strategies (DJ and SD, 2000). It has been reported that ROS generated by PDT could induce both autophagy and apoptosis in cancer cells. Pretreatment with autophagy inhibitor decreased the ratio of LC3 II/LC3 I, a standard autophagy marker, suggesting DVDMS-PDT initiated autophagic response in tumor cells. MAPK pathways are also involved in ROS triggered cellular responses. Heme oxygenase-1 (HO-1) is a rate-limiting enzyme that has a special protective effect on cells (So and Oh, 2016). After photo-therapy, phosphorylation of p38 MAPK and JNK was upregulated, the expression of HO-1 was rapidly increased and gradually returned to normal levels; the ROS scavenger further decreased phosphorylation of p38MAPK and HO-1, suggesting increased ROS level post PDT would trigger multiple signals activation and some contribute to the ultimate cell death, while others protect cellular oxidative stress (Wang et al., 2014). Zhu et al. evaluated the role of autophagy in the antitumor process of DVDMS-PDT against human colorectal cancer (Zhu et al., 2019). DVDMS-PDT showed better antitumor efficiency than Photofrin^®^-PDT. Chloroquine (CQ) promoted apoptosis by inhibiting autophagy, suggesting that autophagy may play a protective role in DVDMS-PDT treated cells. Furthermore, no visible tumor cells were found in the CQ+DVDMS-PDT group, which confirmed the hypothesis that autophagy was protective under the experimental conditions.

Surgical removal of the primary tumor is highly effective in many cancer patients. However, survival is noneffective when there are metastatic lesions and no response to treatment (Jr et al., 2011). Therefore, inhibition of metastasis is crucial to the prognosis of tumor therapy. Wang et al. reported that DVDMS-PDT inhibited the invasion capacity and migration of 4T1 cells (Wang et al., 2015b). Many investigations have shown that microvilli on the surface of tumor cell are closely related with cell migration (Hrazdira et al., 1998). Microvilli mediate the exchanges of substances and nutrition and promote the malignant proliferation and attachment of tumor cells. Herein, the microvilli were observed to disappear after DVDMS-PDT treatment under scanning electron microscope. Moreover, the F-actin cytoskeleton is a critical structural network that affects cell contraction, movement, and vesicular transport (Furukawa et al., 2013). DVDMS-PDT interferes with the regular pattern of actin filaments in a light-dose dependent manner, suggesting that the collapse of actin network was involved in cell migration and invasion (Wu et al., 2016). This is consistent with previous report that PDT may cause damage to the cell surface or cytoskeleton, thus produce inhibitory effects on cell proliferation and motility (Pacheco-Soares et al., 2014). Hu et al. compared the metastasis suppression between DVDMS-PDT and Photofrin^®^-PDT in 4T1 xenograft model, showing better efficiency in the former than in the latter.

Moreover, PDT belongs to nonionizing irradiation and can be administered repeatedly without causing long-term complications. Clinical and experimental results have proved that repeated application of PDT has effectively improved the therapeutic effect (Togsverd‐Bo et al., 2015). Xiong et al. designed different regimens to explore the optimal therapeutic effect of DVDMS-PDT, suggesting one injection of DVDMS followed by three time laser exposure within a special intervals exhibited superior inhibition for tumor growth, angiogenesis and metastasis (Xiong et al., 2016).

### DVDMS-Based Sono-Therapy

**Figure 2:**
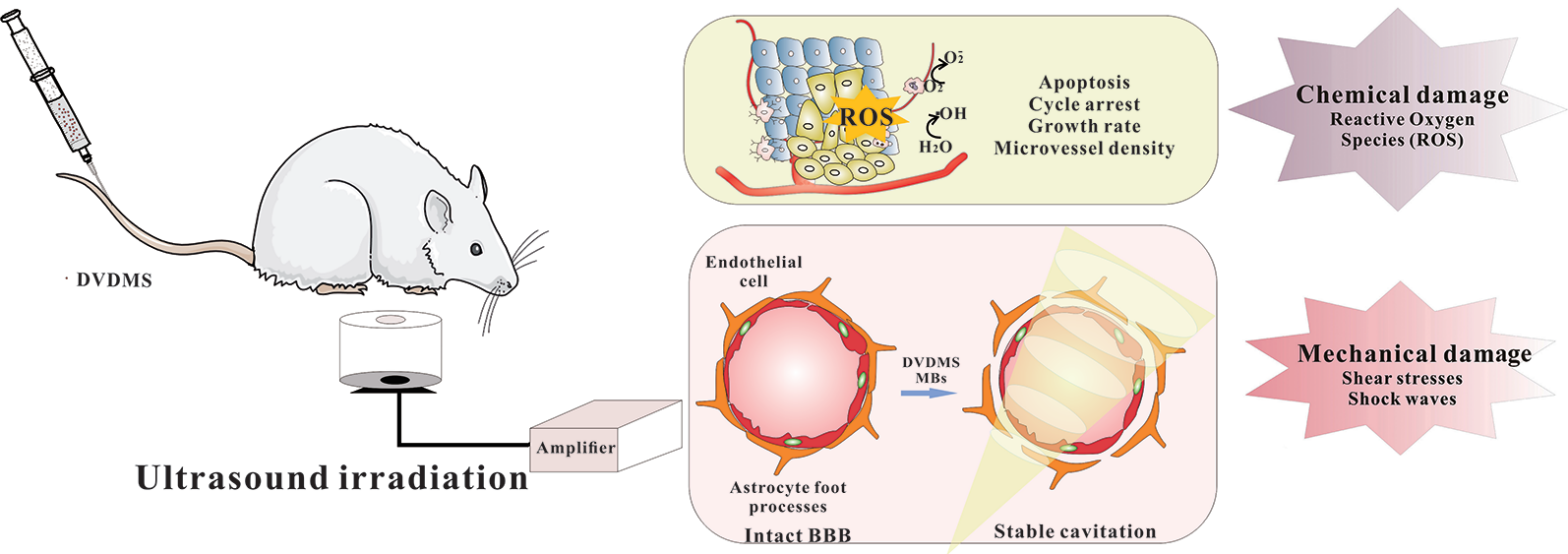
The possible mechanisms of DVDMS-SDT.

Sono-therapy based on the synergy of sensitizer and ultrasound, involves several mechanical, chemical, and cavitational activated mechanisms (**Figure 2**). Shen et al. explored the antitumor effect of DVDMS-SDT on the human small lung cancer (Shen et al., 2018). DVDMS-SDT increased cellular apoptosis, ROS levels as well as cleaved caspase-3, -8, -9, and -10, decreased the levels of MMP, RIP3, Bcl-2, VEGF, and TNF-α, suggesting DVDMS-SDT induced H446 cells apoptosis in part by mitochondria-dependent signaling pathway and the extrinsic apoptosis was also involved. This is the first study to provide evidence that RIP3 expression was inhibited by DVDMS-SDT in H446 cells. The antitumor effect of DVDMS-SDT is generally ROS-apoptosis dependent Li et al. studied the antitumor effect of DVDMS-SDT on hepatocellular carcinoma cell lines (Li et al., 2019). The results indicate that DVDMS-SDT was more effective than PpIX-SDT in inhibiting the growth of HepG2 cells, accompanied by the increased enrichment of DVDMS in cells and G2/M phase arrest with decrease of CDK1 and Cyclin B1. Furthermore, the increased of ROS level can up-regulate the expression of p53 and Bax, down-regulate the expression of Bcl-2, leading to the activation of caspase-3 and ultimate initiation of cell apoptosis. Besides, ultrasound is relatively repeatable, easily accessible, inexpensive, and nontoxic, the multiple exposure of DVDMS would be more efficient against tumors. Xiong et al. monitored a significant proliferating cell nuclear antigen (PCNA) drop after DVDMS plus multiple ultrasound treatments compared with either mono-treatment. Such multiple SDT also significantly reduced the microvessel density (MVD) and tumor growth of xenografts (Xiong et al., 2015).

Microbubbles (MBs) are widely used as contrast agents in medical diagnosis (Dijkmans et al., 2004). The shock waves generated by MBs during the sound pressure process improve the permeability of cell membranes by transient sonoporation (Rapoport et al., 2009). Based on this mechanism, MBs has been widely used in drug delivery in recent years (Yang et al., 2014). With the development of SDT, MBs are introduced to enhance its curative effect (Roberto et al., 2013). SonoVue^®^ is a clinically-approved MB, and currently widely used in clinic application (Nicolau et al., 2004). Wang et al. investigated the associated effects of DVDMS-SDT and SonoVue^®^, and the findings suggest that the existence of MBs promoted the transient transportation and internalization of DVDMS, which further enhanced the cytotoxicity of ultrasound. Ultrasound-targeted microbubble destruction (UTMD) combines the advantages of ultrasound diagnosis, therapeutics, and spatiotemporal controllable-platform, gradually becoming a theranostic strategy in various diseases. The use of UTMD to promote the accumulation of sensitizers in the lesions, is considered to be one of the ways to improve the antitumor activity of SDT (Wang et al., 2015a). Pi et al. investigated the antitumor effect of DVDMS-SDT on intracranial human glioblastoma in nude mice (Pi et al., 2019). Longitudinal bioluminescence imaging showed that the growth of intracranial glioblastoma was slower in SDT group. The median survival time was prolonged to 30.25 days after SDT treatment, indicating that DVDMS-SDT with the help of MBs provides a new promising therapeutic strategy against glioblastoma. Pi's study also shows UTMD caused significantly enhanced delivery of DVDMS, including apoptosis increase as well as cell proliferation suppression. These indicate that MBs combined with DVDMS-SDT may be a promising treatment for human glioblastoma.

### The Combined Strategy for Enhanced Sonoeffects

#### The Combination of SDT With PDT

Sono-PDT (SPDT) is a new combination of SDT and PDT for the treatment of cancer (Jin et al., 2000). Previous studies have shown that SPDT can significantly enhance the antitumor activities against of various malignants at preclinical and clinical levels. From the perspective of physical properties of ultrasound, enhanced therapeutic effects may involve ROS formation, thermal effect, mechanical stress, cavitational effect and other factors (Hirschberg and Madsen, 2017). Liu et al. examined the effects of DVDMS-SPDT, the findings show that: (1) compared with either monotherapy, combined therapy significantly enhanced tumor inhibition; (2) SPDT achieved a more efficient outcome even utilizing a much lower PDT dose, which may be due to subsequent SDT could compensate for the inevitable attenuation of PDT upon reaching deep tissues; (3) excessive ROS contributes to the enhanced anticancer efficiency of the combination; (4) ultrasound increased permeability of cell membrane and thus induced improvement of cellular uptake of DVDMS. Based on the results, the combination of PDT with subsequent SDT would be a good treatment option. High ROS levels play a key role in cell death. And ultrasound-induced cavitation effects and changes in membrane permeability also contribute to the enhancement of combined therapy (Liu et al., 2016). Because of the complicated system of SPDT, its mechanisms have not been clearly revealed. Further preclinical studies and clinical trials are needed to verify and improve SPDT.

#### The Glycolysis Blockage Aggravated SDT Effects

Changes in the basic characteristics of cancer are interwoven with the intrinsic metabolism of cancer cells (Romero-Garcia et al., 2011). Different tumor cells have different metabolic phenotypes. Glycolysis and mitochondrial oxidative phosphorylation may coexist at the same time, and the mutual conversion also called energy reprogramming happens frequently and differently under distinct treatment conditions (Moreno-Sa´nchez et al., 2007).

Unlike most normal cells, tumor cells have an increased metabolic requirement for glycolysis, also known as the Warburg effect, which generally helps promote metastasis and inhibit apoptosis (Vander Heiden et al., 2009; Cairns et al., 2011). Therefore, the improvement of glycolysis level makes it possible to target tumor metabolism to combat cancer. 2-deoxyglucose (2DG) is the most commonly used antiglycolytic drug. When 2DG enters malignant tumor cells, it is phosphorylated by hexokinase II and inhibits the generation of ATP (Feng et al., 2015). Nevertheless, due to the systemic toxicity of 2DG, it has not been satisfactory in clinical trials for some cancer treatments (Maschek et al., 2004). Xie et al. reported that using SDT and 2DG to target mitochondria and aerobic glycolysis simultaneously was more effective than either alone against breast cancer both *in vitro* and *in vivo*. 2DG regulates cell viability through the metabolic enzymes that affected by ROS generation, and ROS is one of the determinants of SDT, so the synergistic action between 2DG and DVDMS-SDT may converge on ROS. Intracellular ROS levels in SDT+2DG groups was increased approximately 2-fold and 30-fold than that in SDT and 2DG groups, respectively. The cellular oxygen consumption rate (OCR) level and ATP generation decreased after 2DG treatment, especially in SDT. Therefore, it can be speculated that the combination of SDT and 2DG significantly suppressed oxidative phosphorylation and glycolysis, leading to ATP depletion. Besides, SDT increases the sensitivity of tumor cells to 2DG treatment by blocking the energy replenishment pathways. Ultimately, the tumor inhibition rate of the combined therapy was higher than the monotherapies (Xie et al., 2018). The findings indicate that SDT+2DG could provide a new noninvasive treatment for high mortality metastatic tumors.

## DVDMS-Loaded Nanoparticles for Precision Theranostics

**Figure 3:**
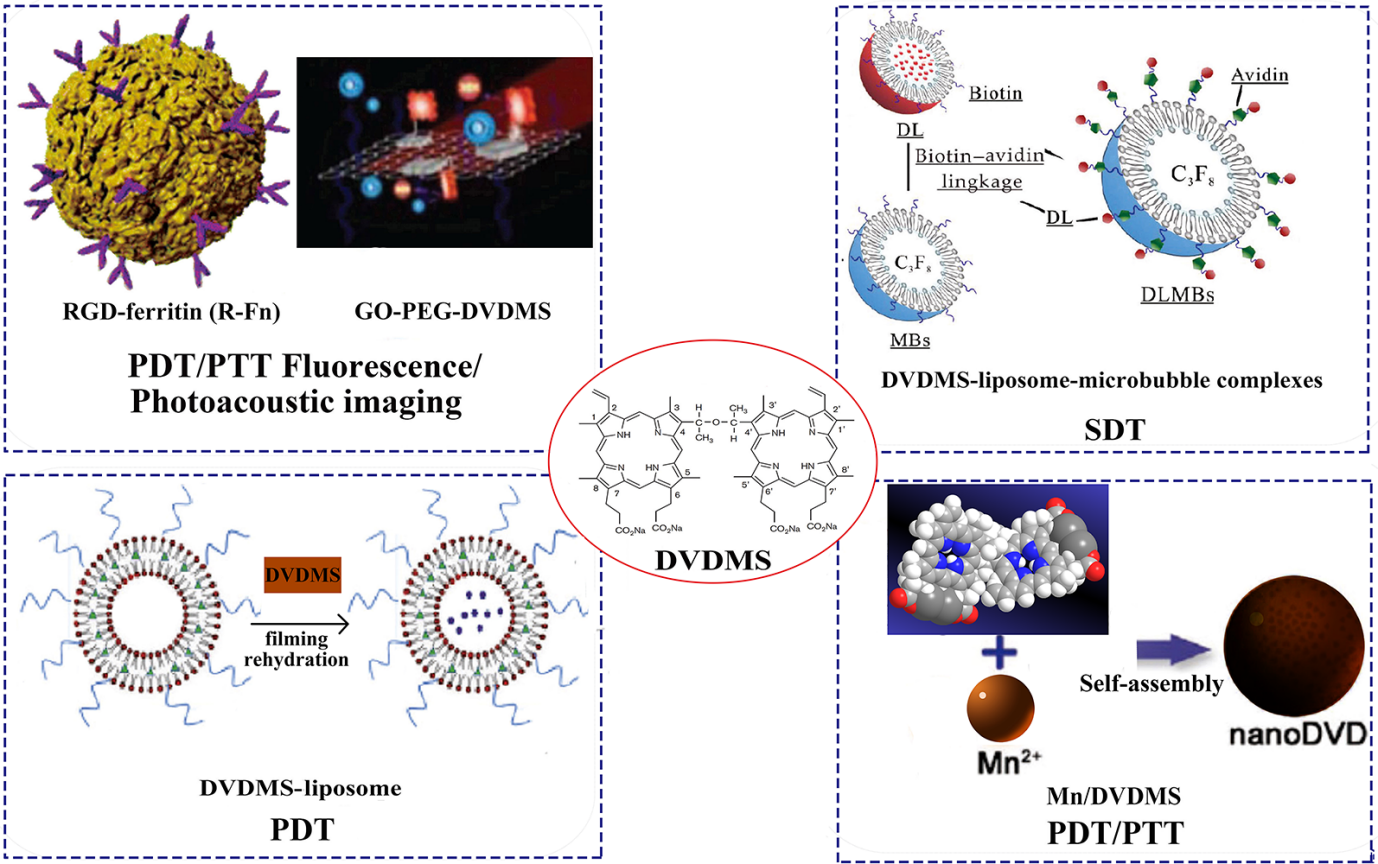
Different DVDMS nanoparticles of photo-/sono-therapy and image-guided cancer treatment. Reprinted with permission of ref. (Yan et al., 2014), (Huang et al., 2017), Royal Society Of Chemistry. (Liu et al., 2018), WILEY.

We summarize the different nanoDVDMS used in photo-/sono-therapy and imaging-guided modality for cancer treatment, such as liposomes, micro-/nano-bubbles, graphene oxide, metal NPs (**Figure 3**).

### NanoDVDMS for Imaging Guided Photo-Therapy

In order to improve the controllability of drug release, a variety of nanoscale drug delivery systems (DDS) have been developed. Light-responsive DDS attract great interest because of its easy application and spatiotemporal performance (Pashkovskaya et al., 2009). Wang et al. developed a dual-effect liposome with coencapsulation of DVDMS and an antimitotic agent (PTX). Both *in vivo* and *in vitro* studies have confirmed that the compound liposomes had excellent anticancer activity through the synergistic effect of PDT and PTX cytotoxicity, and the DVDMS fluorescence guided photo-therapy exhibited minimal side effects. As a ROS production promoter, DVDMS enhanced the release of embedded PTX and enhanced the efficiency of chemotherapy by reducing the core molecule McL-1, thereby activating apoptosis. DVDMS-PDT treatment reduced cellular glycolysis, thus preventing the possible energy conversion and struggling survival after PTX treatment. Therefore, apoptosis sensitivity is the main reason for laser-irradiated liposome nanoDVDMS to enhance PTX sensitivity of cells (Wang et al., 2017). Sun et al. used iRGD modified DVDMS liposome (iRGD-Lipo-DVDMS) combined with UTMD to open the BBB and targeted glioma therapy (Sun et al., 2019). The results show that iRGD-modified liposomes improved the targeting ability of tumors compared with the liposomes without iRGD. The iRGD-Lipo-DVDMS exhibited significantly improved drug accumulation in monolayer cells, 3D tumor spheroids and transplanted C6 tumors, and significant apoptosis in glioma cells after combined with SDT treatment. In addition, the developed nanosonosensitizers have good biocompatibility *in vivo* and have broad prospects in fluorescence image-guided sonodynamic tumor therapy. Taken together, iRGD-Lipo-DVDMS can provide targeting ultrasound actived DDS and would become an alternative strategy for glioma treatment.

As an inorganic nanocarrier, graphene oxide (GO) has many advantages, such as abundant functional groups and easy surface modification (Tang et al., 2012). Chen et al. designed a novel phototheranostic nanoplatform based on DVDMS-loaded PEGylated GO (GO-PEG-DVDMS). In this study, GO-PEG enhanced the tumor accumulation efficiency of DVDMS and fluorescence imaging-guided PDT, showing a strong antitumor effect (Yan et al., 2015). Further, they strategically designed another nanotheranostic platform based on GO-PEG-DVDMS for enhanced fluorescence/photoacoustic (PA) dual-modal imaging (Yan et al., 2014). The GO-PEG carrier greatly improved the fluorescence characteristics, PA imaging and PTT of DVDMS through intramolecular charge transfer and near infrared (NIR) absorption. The treatment effect of GO-PEG-DVDMS is significantly better than that of PDT or PTT alone. In another report, DVDMS were loaded into RGD-modified ferritin nanoparticles for image-guided PDT/PTT combination therapy. The loading capacity of DVDMS in the prepared nanocomposites was up to 66.67 wt%, and the tumor treatment effect was significantly better than that of DVDMS (Huang et al., 2017).

In addition, photosensitizers in photo-therapy are often limited by photobleaching and tumor hypoxia. Through supramolecular assembly, the researchers further developed the coordinated assembly strategy of tumor environmental trigger to form Mn/DVDMS nanotheranostics for cancer photo-therapy (Chu et al., 2017). In MCF-7 cells and xenograft tumors, MnO_2_/DVDMS was reduced by glutathione (GSH) and H_2_O_2_ and reassembled into nanoDVDMS, which can be monitored by activated magnetic resonance/fluorescence/photoacoustic signals. Interestingly, the reduction of GSH, the generation of O_2_ and the formation of nanoDVDMS have synergistic effects with photo-therapy, which have improved the antitumor efficacy and provided a new approach for tumor treatment.

Zang et al. designed Gd-DVDMS as a water-soluble and multifunctional theranostic agent as it can serve as a PS in PDT, a phosphorescence-based oxygen indicator, and a magnetic resonance imaging contrast agents (Zang et al., 2017b).

### DVDMS-Loaded Nanoparticles for Enhanced Sono-Therapy

MBs can be used as potential delivery carriers. The therapeutic agents can be coadministrated with MBs in a variety of ways: drugs that co-injected together with MBs; drugs that encased in MBs casings/cavities; drugs that covalently linked at the shell surface of MBs; drugs that encapsulated in nanoparticles and then linked with MBs (Heleen et al., 2015; Zhang et al., 2017). Based on the sonoactivated features of DVDMS, Li et al. designed the liposome-encapsulated DVDMS with microbubbles *via* biotin-avidin linkage, resulting in a complex called DLMBs. Compared with free or liposome DVDMS, DLMBs has better ultrasonic cytotoxicity to breast cancer. Both *in vivo* and *in vitro* studies have confirmed that DLMBs combined with ultrasonic therapy has significant antitumor activity. The active substance induced by ultrasound was the key mediator that triggers the increased release of DVDMS in DLMBs, promotes cellular uptake and intratumor diffusion, and enhances sonotoxicity of DVDMS (Li et al., 2018).

Liu et al. reported a multifunctional theranostic agent that integrate imaging and therapy into a single nanoplatform for MR and fluorescence image-guided SDT treatment. SDT reagents were prepared by encapsulating DVDMS chelating with manganese ions into nanoliposomes (DVDMS-Mn-LPs) (Liu et al., 2018). Both cell and animal studies demonstrated that SDT combined with DVDMS-Mn-LPs significantly improved the antitumor growth efficiency. In addition, DVDMS-Mn-LPs are good for MR and fluorescence imaging. Therefore, DVDMS-Mn-LPs may provide a promising strategy for imaging-guided modality for cancer treatment.

## DVDMS Used in Antibacterial Application

In terms of nononcology disease research, the abuse of antibiotics has caused a growing problem of bacterial resistance (Costerton et al., 1999). Mai et al. investigated the effect of DVDMS-PACT of *Staphylococcus aureus* (*S. aureus*) and Multidrug-resistant (MDR)-*S. aureus* (Mai et al., 2016; Mai et al., 2017). The results show that DVDMS-PACT decreased the survival of bacteria planktonic and biofilm culture. PACT treatment produces a large amount of reactive oxygen species. Furthermore, the DNA damage and membrane permeability after DVDMS-PACT were significantly increased, which might be crucial for PACT efficient outcome. Studies demonstrate that *S. aureus*/MDR- *S. aureus* could rapidly photoinactivate in wound infection when it is treatment with DVDMS-PACT. Moreover, the levels of inflammatory cytokines decreased and growth factors increased after PACT treatment at wound sites, suggesting that the treatment can inhibit wound deterioration, reduce inflammation and promote wound healing. There are no detectable side effects using DVDMS-PACT at the therapeutic dose according to the preliminary safety analysis.

## Conclusion and Outlook

DVDMS has good physical and chemical properties, which would be a valuable reference for further investigation of DVDMS mediated activated cancer therapy. DVDMS-triggered sono-/photo-therapy produces significant efficiency both *in vitro* and *in vivo*, and numerous signal transduction and cell death pathways are also involved in the oxidization stress and antioxidant processes during SDT/PDT. Besides, DVDMS-PACT can greatly suppress bacteria and MDR-bacteria proliferation, and promote the healing of wounds in burn infection. Moreover, with the development of nanotechnology, various nanoDVDMS has been designed to provide better biocompatibility, physiological stability, excellent targeting, enhanced sono/phototoxicity, and integration of diagnostic and therapeutic features. These novel theranosic nanoplatforms hold great promise for precision recognition and treatment of malignant tumor as well as other diseases, which could be expected to be applied in future clinical translation.

## Author Contributions

PW and XW contributed design of this review. BM, QL, and KZ organized the literatures and wrote the draft of this manuscript. BM, XW, and PW contributed to manuscript revision. All authors read and approved the submitted version.

## Funding

This research was supported by the National Natural Science Foundation of China (Grant No. 81872497 and No. 81972900) and the Fundamental Research Founds for the Central Universities (2017TS039).

## Conflict of Interest

The authors declare that the research was conducted in the absence of any commercial or financial relationships that could be construed as a potential conflict of interest.

The handling editor is currently organizing a Research Topic with one of the authors, XW, and confirms the absence of any other collaboration.

The reviewer WT declared a shared affiliation, with no collaboration, with the authors to the handling editor at time of review.
